# Responses of Ecological Stoichiometric Characteristics of Carbon, Nitrogen, and Phosphorus to Periodic Submergence in Mega-Reservoir: Growth of *Taxodium distichum* and *Taxodium ascendens*

**DOI:** 10.3390/plants10102040

**Published:** 2021-09-28

**Authors:** Dongdong Ding, Minghui Liu, Muhammad Arif, Zhongxun Yuan, Jiajia Li, Xin Hu, Jie Zheng, Changxiao Li

**Affiliations:** Key Laboratory of Eco-Environments in the Three Gorges Reservoir Region (Ministry of Education), Chongqing Key Laboratory of Plant Resource Conservation and Germplasm Innovation, College of Life Sciences, Southwest University, Chongqing 400715, China; dingdongdong@email.swu.edu.cn (D.D.); shengtai2017@email.swu.edu.cn (M.L.); muhammadarif@swu.edu.cn (M.A.); yuanzhongxun@email.swu.edu.cn (Z.Y.); ljj133888@email.swu.edu.cn (J.L.); sthuxin@email.swu.edu.cn (X.H.); jiezheng@email.swu.edu.cn (J.Z.)

**Keywords:** Three Gorges Reservoir, riparian belt, hydro-fluctuation zone, submergence, conifers

## Abstract

Ecological stoichiometric studies can be useful for managing the deteriorated riparian zones of mega-reservoirs in which nutrients significantly impact the balanced vegetation cover. The present study aims to explore the effects of periodic submergence on the stoichiometric ecological characteristics of carbon (C), nitrogen (N), and phosphorus (P), as well as the growth conditions of two leading conifer species (*Taxodium distichum* and *Taxodium ascendens*) in the hydro-fluctuation zone of the Three Gorges Reservoir (TGR) region, China. The stoichiometrical contents of C, N, and P in fine roots, leaves, and branches, and the growth conditions of *T. distichum* and *T. ascendens* were measured in July 2019. The results showed that periodic submergence affected the stoichiometric characteristics and growth conditions of these two woody species, and the impact was restrained, but both grew well. The effects of inundation on the C, N, and P ecological stoichiometric characteristics differed in different parts of trees. In general, the C contents showed the following pattern: leaves > branches > fine roots. The N and P content showed the following pattern: leaves > fine roots > branches, while the C/N and C/P ratios showed an opposite trend to that of N and P. The N and P content in all parts of *T. distichum* (with means of 17.18 and 1.70 g/kg for leaves, 4.80 and 0.57 g/kg for branches, and 6.88 and 1.10 g/kg for fine roots, respectively) and *T. ascendens* (with means of 14.56 and 1.87 g/kg for leaves, 5.03 and 0.63 g/kg for branches, and 8.17 and 1.66 g/kg for fine roots, respectively) were higher than the national average level (with means of 14.14 and 1.11 g/kg for leaves, 3.04 and 0.31 g/kg for branches, and 4.85 and 0.47 g/kg for fine roots, respectively). Except for N and P contents in the leaves of *T. distichum*, there was a significant correlation between N and P elements in other parts (*p* < 0.05). Nevertheless, the N/P ratio (10.15, 8.52, 6.44, and 7.93, 8.12, 5.20 in leaves, branches, and fine roots of *T. distichum* and *T. ascendens*, respectively) was lower than the critical ratio of 14. The growth conditions of *T. distichum* and *T. ascendens* were significantly negatively correlated with their leaf C contents and significantly positively correlated with their fine root N and P contents. This study showed that *T. distichum* and *T. ascendens* could maintain their normal growth needs by properly allocating nutrients between different organs to adapt to the long periodic submergence in the hydro-fluctuation zone of the TGR region.

## 1. Introduction

The Three Gorges Dam was constructed for the purposes of hydropower generation, flood control, and navigation, but it has disrupted the natural dynamic balance of the upper reaches of the Yangtze River and formed the Three Gorges Reservoir (TGR) [1,2]. As a consequence of water-level management, the water level of the TGR fluctuates annually from 145 m above sea level (a.s.l.) in summer (i.e., from May to September) to 175 m a.s.l. in winter (i.e., from October to the following April) [3,4]. This results in forming a hydro-fluctuation zone with a total area of 350 km^2^ with a 30 m variation in elevation [5]. Due to the use of the “winter storage and summer drainage” water transfer mode, this anti-seasonal water transfer mode has had a huge negative effect on the riparian ecosystem. In the hydro-fluctuation zone, plants suffered from serial submergence stress with durations as long as 210 days at depths of up to 30 m [6]. Prolonged submergence extirpated the original vegetation and caused many ecological and environmental problems, such as soil erosion [7], land-use challenges, and non-point-source pollution [8]. As a result, many native plants die out because they cannot adapt to this change, and vegetation in the water-fluctuation zone is seriously affected and degraded [3]. Since vegetation plays a crucial role in maintaining biodiversity and ecosystem stability in the water-level fluctuation zone of the TGR [8], vegetation restoration in the water-level fluctuation zone has attracted extensive attention in recent years. Vegetation reconstruction and restoration in the newly formed hydro-fluctuation zone is critically important for ecological safety and restoration of the anthropogenically impacted ecosystem there. Exploring suitable plants’ physiological and ecological adaptation mechanisms is a critical foundation for vegetation restoration and the premise for resolving ecological and environmental problems in the TGR’s water-level fluctuation zone and other similar degenerative riparian areas and stream ecosystems. Previous studies have screened out some flood-tolerant plants, and *Taxodium distichum* and *Taxodium ascendens* have been selected as excellent tree species for vegetation reconstruction in the water-level fluctuation zone because of their rapid growth and strong adaptability [9,10,11].

The growth, photosynthetic response, physiological metabolism, and nutrient levels of *T. distichum* and *T. ascendens* under flooding stress have been evaluated using in situ and controlled garden experiments [4,10,12], which better explain the flooding tolerance mechanism of *T. distichum* and *T. ascendens*. However, under flooding stress conditions, many physiological processes related to plant C, N, and P can be constrained, such as C-sequestration, N-fixation, and nutrient mineralization. The balance of nutrient elements in interactions and processes is known as ecological stoichiometry. Ecological stoichiometry is an important tool for studying ecological processes and functions, which is used to explore the dynamic balance of various elements and their interactions, as well as the limitations on plant growth [13,14]. Thus, as a result of years of in-site flooding, the C, N, and P ecological stoichiometric characteristics of *T. distichum* and *T. ascendens* deserve more attention, as they are the leading tree species in the area. C, N, and P are essential elements of plant bodies, and C, N, and P ecological stoichiometry represents an organism’s demand for natural resources and connects different levels of biogeochemical cycling; furthermore, their changes affect the growth and development of plants [15] and the evolution of plant community structure [16]. Studies on the contents and ratios of C, N, and P in plants can help solve the balance between supply and demand for nutrients in plants and ecosystems, as well as understand the nutrient cycling process and the survival strategies formed by plants adapting to environmental changes [17]. This will be helpful to further understand the mechanisms of flooding tolerance and promote the restoration and reconstruction of degraded vegetation in the water-level fluctuation zone.

Plant ecological stoichiometry has been widely used in the study of plant adaptation mechanisms and nutrition strategies under stressed environments, mainly including the characteristics of biological nutrient allocation, cycle, supply, and demand in the forest [18], desert [19], grassland [20], and wetland [17,21], as well as their relationship with habitat. In addition, studies on the ecological stoichiometry characteristics of plants in different ecosystem types have been carried out at the global [22,23], national [16], and regional [24] scales, which further promote the development of ecological stoichiometry. However, previous studies on plant nutrient stoichiometry have mainly focused on a single organ, especially green or senescent leaves [23,25,26]; few studies have investigated the nutrient stoichiometry of branches and roots. Some studies have found that branches and roots are structural and transport organs, in addition to playing a role in nutrient acquisition and storage [27]. Nutrient stoichiometry and allocation patterns in different plant organs (e.g., leaves, branches, and roots) reflect the tradeoffs a plant faces in drawing aboveground and belowground resources [28]. Therefore, to study the response of plants to the environment, each plant organ must be considered simultaneously [29]. Leaves, branches, and fine roots are the three important organs of plants, which play different roles in the growth process of plants. N and P are required in leaves for metabolism, and woody branches store C, N, and P [30], whereas fine roots are the primary organs for plants to absorb nutrients directly from the soil [31]. The division of labor among the three parts is clear and closely related, which is essential for plant growth and internal nutrient cycling. Therefore, studying the C, N, and P ecological stoichiometry characteristics of plant leaves, branches, and fine roots will help us better understand the survival strategies of plants and, with great significance, reveal the ecological strategies and environmental adaptability of plants under the influence of anti-seasonal water-level fluctuations.

The present study focuses on the patterns of C, N, and P stoichiometry among plant organs and the growth conditions of *T. distichum* and *T. ascendens*. The current study also determines their responses to periodic submergence in the hydro-fluctuation zone of the TGR region. Thus, we hypothesized that (1) both species have strong flooding tolerance, but high-intensity flooding would inhibit plant growth to some degree, (2) the growth was significantly correlated with the C, N, and P content of the two species, and (3) the two species may have different adaptation strategies to the periodic submergence environment in the hydro-fluctuation zone of the TGR region. This study could improve the theory of ecological stoichiometry and provide the basis for vegetation protection and restoration in the hydro-fluctuation zone of the TGR.

## 2. Results

### 2.1. Effects of Submergence Treatment on the Growth

After experiencing seven annual cycles of periodic submergence, the growth of plants in the three submergence treatment groups showed certain differences (Table 1), and their growth trend was as follow: DS (deep submergence) group < MS (moderate submergence) group < SS (shallow submergence) group. With the increase in submergence depth, the plant height, crown area, basal diameter, and DBH of *T. distichum* were significantly restricted (*p* < 0.05, Table 1). The changing trend in plant height and crown width of *T. ascendens* was consistent with *T. distichum*. However, compared to the basal diameter and DBH of *T. ascendens* in MS, there was no significant difference between SS and DS. Nevertheless, the DS group was significantly lower than the SS group (*p* < 0.05).

### 2.2. Effects of Submergence Treatment on the C, N, and P Ecological Stoichiometric Characteristics

According to the results of two-way ANOVA, except for C, the contents of *T. distichum* were not affected by different submergence treatments (*p* > 0.05). The C, N, and P contents and stoichiometric ratio of *T. distichum* were significantly affected by different submergence treatments, different organs, and their interaction (*p* < 0.01) (Table 2). Except for the P contents of *T. ascendens*, which were unaffected by different submergence treatments (*p* > 0.05), and the C contents, which were unaffected by the interaction of different submergence treatments and different organs (*p* > 0.05), the C, N, and P contents, as well as the stoichiometric ratio of *T. ascendens*, were significantly affected by different submergence treatments, different organs, and their interaction (*p* < 0.01) (Table 3).

**Figure 1:**
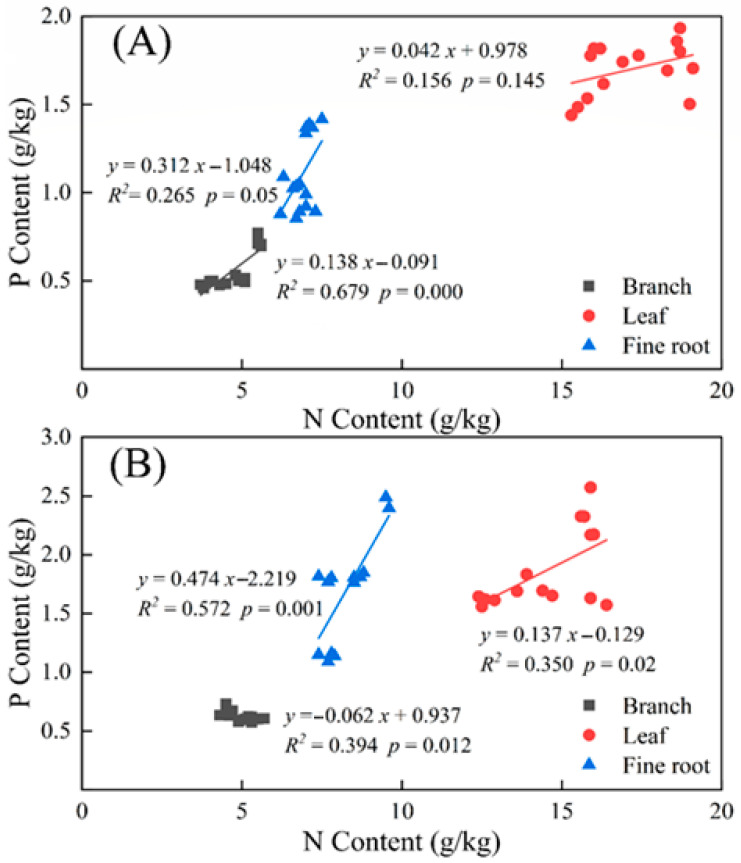
Linear correlation of N and P content in fine roots, leaves, and branches of *Taxodium distichum* (**A**) and *Taxodium ascendens* (**B**); *Y*—the linear correlation equation in which; *r*—the correlation coefficient and *p*—the significance level.

Correlation analysis of N content and P content showed that the N content and P content in branches and fine roots were significantly positively correlated. In contrast, the N and P contents in leaves were not significantly correlated with *T. distichum* (Figure 1A). There was a significant negative correlation between N contents and P contents in branches and a significant positive correlation between N contents and P contents in leaves and fine roots of *T. ascendens* (Figure 1B).

**Figure 2:**
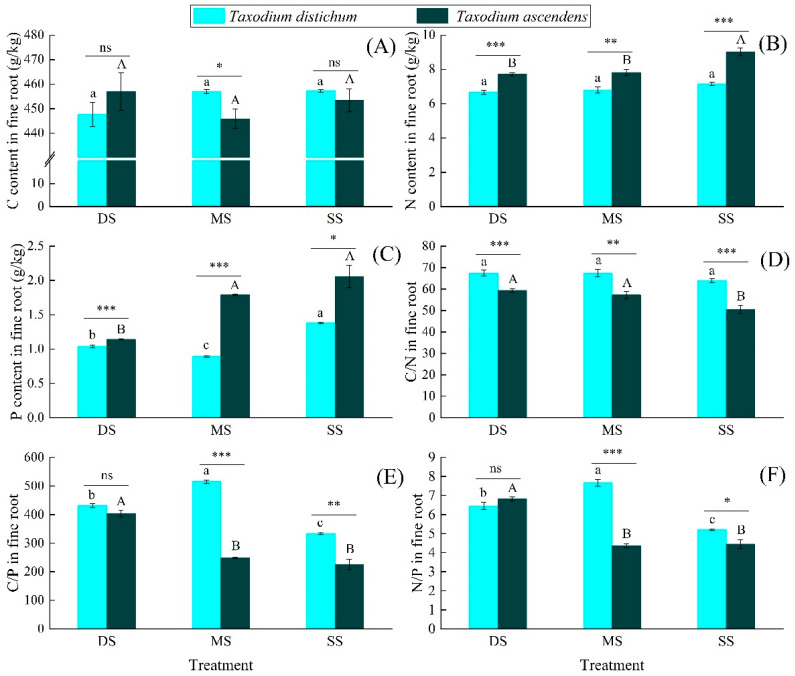
The C, N and P content (**A**–**C**) and C/N/P stoichiometry (**D**–**F**) in the fine root of *Taxodium distichum* and *Taxodium ascendens* under different submergence treatments. Values are means ± SE. Note: According to Tukey’s test, the values with different letters are significantly different at *p* < 0.05. Lowercase letters are used for *Taxodium distichum*, while uppercase letters are used for *Taxodium ascendens*. ***, **, and * indicate significance at the levels of 0.001, 0.01, and 0.05 among different species in the same submergence treatment, respectively; ns means no significant difference.

Flooding significantly reduced the P contents in the fine roots of *T. distichum* and the N contents in the fine roots of *T. ascendens* (*p* < 0.05), whereas it had no significant effects on the N contents in the fine roots of *T. distichum*. The P content in the fine roots of *T. ascendens* in the DS group was significantly lower than that in the MS and SS groups. In all submergence treatment groups, the N and P contents in the fine roots of *T. ascendens* were significantly higher than those of *T. distichum* (*p* < 0.05). In the MS group, the C content in the fine roots of *T. distichum* was significantly higher than that of *T. ascendens* (*p* < 0.05), but the C content in the fine roots of both was not affected by flooding. Therefore, the effects of flooding on the C/N ratio and C/P ratio in fine roots were opposite to the N and P content trend. There was a significant difference in the N/P ratio in the fine roots of *T. distichum* among different submergence treatment groups (*p* < 0.05). With the increase in submergence depth, it showed a trend of first increasing and then decreasing. There was no significant difference in the N/P ratio in the fine roots of *T. ascendens* between the MS and SS groups. Nevertheless, they were significantly lower than that of the DS group (*p* < 0.05) and substantially lower than that of *T. distichum* (*p* < 0.05) (Figure 2).

**Figure 3:**
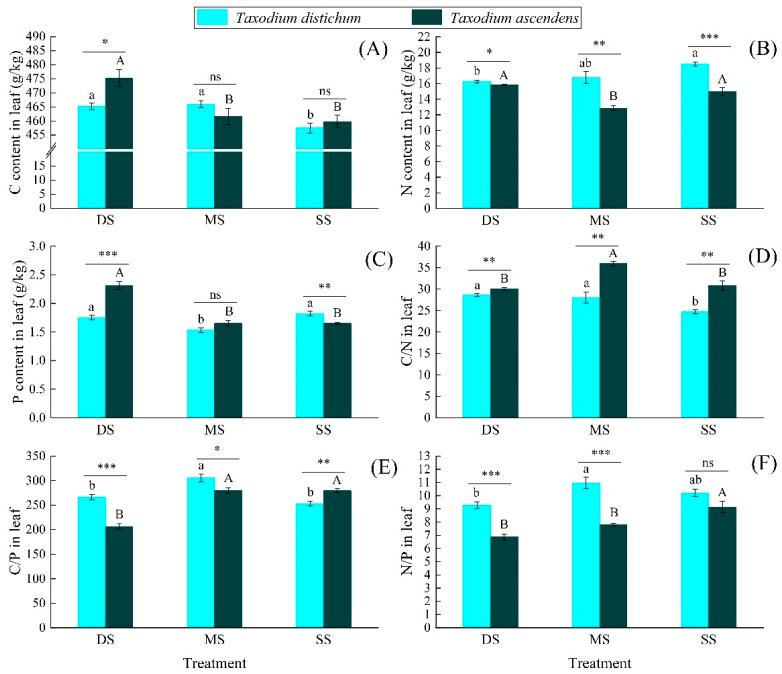
The C, N, and P content (**A**–**C**) and C/N/P stoichiometry (**D**–**F**) in the leaf of *Taxodium distichum* and *Taxodium ascendens* under different submergence treatments. Values are means ± SE. Note: According to Tukey’s test, the values with different letters are significantly different at *p* < 0.05. Lowercase letters are used for *Taxodium distichum*, while uppercase letters are used for *Taxodium ascendens*. ***, **, and * indicate significance at the levels of 0.001, 0.01, and 0.05 among different species in the same submergence treatment, respectively; ns means no significant difference.

As shown in Figure 3, flooding increased the C content significantly in the leaves of *T. distichum* and *T. ascendens*. In the DS group, the C content in the leaves of *T. ascendens* was significantly higher than that of *T. distichum* (*p* < 0.05). With the increase in submergence depth, the N content in the leaves of *T. distichum* significantly decreased. There was no significant difference in the N content in the leaves of *T. ascendens* between the DS and SS groups, but they were substantially higher than those in the MS group (*p* < 0.05). The N content in the leaves of *T. distichum* in all treatment groups was significantly (*p* < 0.05) or extremely significantly (*p* < 0.01) higher than that of *T. ascendens*. The P content in leaves of *T. distichum* in the DS and SS groups was significantly higher than that of the MS group (*p* < 0.05). The P content in *T. ascendens* in the DS group was considerably higher than that of the MS and SS groups. In the DS group, the P content in the leaves of *T. ascendens* was significantly higher than that of *T. distichum* (*p* < 0.001); however, in the SS group, it was substantially lower than that of *T. distichum* (*p* < 0.01). The C/N and C/P ratios in the leaves of *T. distichum* and *T. ascendens* showed opposite trends to the N and P contents, respectively. The N/P ratio in the leaves of *T. distichum* in the MS group was significantly higher than that in the DS group (*p* < 0.05), and the N/P ratio in the leaves of *T. ascendens* in the SS group was considerably higher than that in the DS and MS groups (*p* < 0.05). In the DS and MS groups, the N/P ratio in the leaves of *T. distichum* was significantly higher than that of *T. ascendens* (*p* < 0.001).

**Figure 4:**
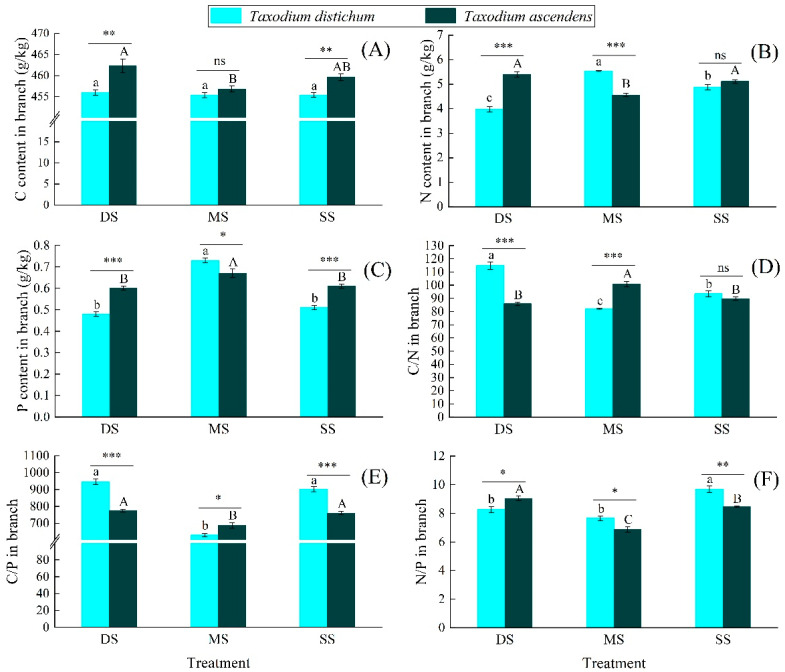
The C, N, and P content (**A**–**C**) and C/N/P stoichiometry (**D**–**F**) in the branch of *Taxodium distichum* and *Taxodium ascendens* under different submergence treatments. Values are means ± SE. Note: According to Tukey’s test, the values with different letters are significantly different at *p* < 0.05. Lowercase letters are used for *Taxodium distichum*, while uppercase letters are used for *Taxodium ascendens*. ***, **, and * indicate significance at the levels of 0.001, 0.01, and 0.05 among different species in the same submergence treatment, respectively; ns means no significant difference.

Different submergence treatments have different effects on the C, N, and P stoichiometric characteristics in the branches of *T. distichum* and *T. ascendens* (Figure 4). There was no significant difference in the C content in the branches of *T. distichum* among different treatment groups. The C content in the branches of *T. ascendens* in the MS group was significantly lower than in the DS group (*p* < 0.05). In the DS and SS groups, the C content in the branches of *T. ascendens* was significantly higher than that of *T. distichum* (*p* < 0.01). The N and P content in the branches of *T. distichum* and *T. ascendens* showed significant differences in different submergence treatment groups. With the increase in submergence depth, the N and P contents in the branches of *T. distichum* and the P content in the branches of *T. ascendens* showed a trend of first increasing and then decreasing. The N content in the branches of *T. ascendens* showed a trend of first decreasing and then increasing. The C/N and C/P ratios in the branches of *T. distichum* and *T. ascendens* showed opposite trends to the N and P contents, respectively. With the increase in submergence depth, the N/P ratio in the branches of *T. distichum* and *T. ascendens* showed a trend of first decreasing and then increasing, and they were significantly different. In the DS group, the N/P ratio in the branches of *T. distichum* was significantly lower than that of *T. ascendens*. At the same time, it was significantly higher than that of *T. ascendens* in the MS and SS groups.

### 2.3. Correlation Analysis of the Growth Conditions and the C, N, and P contents of T. distichum and T. ascendens

Pearson’s correlation coefficients determined the correlations between growth conditions and C, N, and P content. Results showed there were significant correlations between growth conditions and C, N, and P contents. All growth indices of *T. distichum* are significantly positively correlated with fine root N and P contents and leaf N content, and significantly negatively correlated with leaf C content (Table 4). Specifically, for *T. distichum*, plant height, crown area, and DBH were significantly positively correlated (*p* < 0.05) with fine root N content with coefficient values of 0.588 *, 0.516 *, and 0.524 *, respectively. The correlation between plant height, crown area, and DBH and leaf N content was more significant (*p* < 0.01) with peak coefficient values of 0.712 **, 0.685 **, and 0.669 **, respectively. Plant height, crown area, and basal diameter were extremely significantly positively correlated (*p* < 0.01) with fine root P with peak coefficient values of 0.904 **, 0.642 **, and 0.649 **, respectively. Leaf C content was extremely significantly negatively correlated (*p* < 0.01) with plant height (−0.785 **) and crown area (−0.678 **), and significantly negatively correlated (*p* < 0.05) with basal diameter (−0.578 *) and DBH (−0.598 *). DBH was significantly positively correlated (*p* < 0.05) with fine root C content (0.537 *) and branch N content (0.533 *). However, there was some difference in *T. ascendens* (Table 5). Leaf P content was significantly negatively correlated (*p* < 0.01) with plant height (−0.662 **), crown area (−0.687 **), and basal diameter (−0.699 **). The correlation between growth conditions and fine root N content was more significant (*p* < 0.01) in *T. ascendens*, and the highest coefficient values were those of plant height (0.785 **) and crown area (0.697 **).

## 3. Discussion

### 3.1. The Response of Growth to Submergence

Flooding is an environmental stress in many natural and manufactured ecosystems worldwide [32], especially under the special hydrological rhythm of the TGR. In the present research, the growth conditions of both species showed a decreasing trend with the increase in the submergence depth. Nevertheless, they both grew well, which indicated that the two species might actively respond to the water-level change in the hydro-fluctuation zone of the TGR by reducing the biomass allocation of stems and branches [33]. Thus, our first hypothesis that both species have strong flooding tolerance was validated. The survival rate is a critical indicator of plant flooding tolerance [34]. During 7 years of periodic flooding, no mortality was found for the two species, indicating that both species have strong flooding tolerance, closely related to their stable stoichiometric characteristics.

Flooding is a great pressure on plants to survive and grow [35]. Many flooding-tolerant plants may shed dead leaves [36], elongate branches and stems [37], or reduce biomass accumulation when suffering from flooding [38]. These characteristics are critical adaptation strategies to resist anaerobic conditions and maintain growth vigor after emergence from flooding. In the present study, the growth indices of the two species were restricted along with the increase in submergence depth. The flooding induces changes in the plant environment from air to water; hence, plants cannot get enough oxygen [39] and develop low-oxygen quiescence syndrome [32], which slows or inhibits their growth. Moreover, a significant reduction in the crown area may also reduce the total photosynthetic rate and the accumulation of photosynthetic products, thereby slowing the plant’s growth rate. In the SS group, the growth of *T. distichum* was better than that of *T. ascendens* (Table 1), indicating that *T. distichum* could grow better under the condition of sufficient water but no flooding, possibly because the leaf area of *T. distichum* was larger than that of *T. ascendens*, thus increasing the total photosynthetic area so that it could accumulate more photosynthates [10], which confirmed our third hypothesis. However, in the MS and DS groups, the growth of *T. ascendens* was better than that of *T. distichum* (Table 1). The reason may be that *T. distichum* consumes more energy than *T. ascendens* to resist the adverse environment under deeper submergence conditions, and *T. ascendens* shows stronger resilience than *T. distichum*. In addition, previous studies [10] found that, under flooding conditions, the net photosynthetic rate of *T. distichum* was significantly reduced, while the net photosynthetic rate of *T. ascendens* was unaffected or even increased, indicating that *T. ascendens* may have a stronger resistance to flooding than *T. distichum*, which may also explain why *T. ascendens* is growing better.

### 3.2. The Responses of C, N, and P Ecological Stoichiometry Characteristics to Submergence

The C, N, and P contents and their stoichiometric ratios of *T. distichum* and *T. ascendens* were affected by flooding. The effects were different for different organs and element types. On the whole, the element content in the fine roots, leaves, and branches of *T. distichum* and *T. ascendens* in all submergence treatment groups showed the following pattern: C > N > P. The difference confirmed that different plant organs have different physiological functions and nutrient absorption and accumulation capacities [19]. The C contents of the two species were in the order of leaves > branches > fine roots. Although the C content of various organs is different, the ratio of C contents in fine roots, branches, and leaves in different treatments was close to 1:1:1, indicating that the allocation of C in different organs is relatively balanced between the two species. In addition, because the C element in plants mainly comes from the atmosphere, it is less affected by flooding and, thus, is stable.

Element concentrations (especially N and P) are closely related to organ functions [30]. Different structural substances constitute different organs in plants, and the N and P contents in different structural substances are also different [40]. Therefore, the N and P contents in different organs in the same plant are also different [41]. From the perspective of ecology and evolution, the nutrient distribution pattern among different plant’s organs is closely related to their corresponding functional traits [42]. Different organs have different nutrient requirements as organs with rapid growth and active metabolism [43], and the expression of N and P content in the two species showed the following pattern: leaves > fine roots > branches. Compared with leaves, branches and fine roots have relatively low N and P contents because their main functions are to absorb and transport water and nutrients to leaves. The N and P contents in fine roots and leaves of *T. distichum* showed similar changing trends in different submergence treatment groups (Figure 3 and Figure 4), reflecting the integrity of the growth and metabolism process of plants, as well as the consistency of the proportion of photosynthate and nutrient allocation between the aboveground and underground parts [44]. However, the conditions were different in *T. ascendens*. With the increase in submergence depth, the N and P contents in the fine roots of *T. ascendens* gradually decreased, while the N and P contents in the leaves gradually increased. This phenomenon indicates that N and P elements absorbed by *T. ascendens* fine roots are more likely to be transported to leaves [45], enabling rapid metabolism and adequate photosynthesis of leaves [46], and allowing for healthy growth of *T. ascendens*. Hence, our third hypothesis was partly verified.

The ratio of C, N, and P in plants can reflect the adaptation of plants to the environment in which they live [47]. In general, the growth of plants will not be restricted by the C element, and the ratio of C/N and C/P can reflect the utilization efficiency of N and P of plants to a certain extent [48]. The C/N and C/P ratios of *T. distichum* and *T. ascendens* in each submergence treatment group showed the following pattern: branches > fine roots > leaves, indicating that *T. distichum* and *T. ascendens* have higher N and P utilization efficiency in the hydro-fluctuation zone of the TGR region. N and P are the main limiting elements for plant growth, and the N/P ratio can characterize the nutrient limitation of plants. When plant N/P < 14, it means that the N element mainly restricts plant growth; when N/P > 16, it means that the P element mainly determines plant growth; when 14 < N/P < 16, it means that plant growth is subjected to the common limitation of N and P elements [49]. In the present study, the N/P ratios in fine roots, branches, and leaves of *T. distichum* and *T. ascendens* were all less than 14 in all treatments, indicating that the N element may restrict the growth of the two species. However, the concentrations of N and P were higher, except for the N content in the leaves of *T. ascendens*, in the MS group. The N and P contents in branches, leaves, and fine roots in all treatment groups were higher than the national average level [16]. The high N and P concentrations make up for the limitation of the N element [50], which is why the two species can grow healthily in the hydro-fluctuation zone of the TGR region [51]. From the perspective of stoichiometry, when the N and P concentration in the photosynthetic tissue is high, the low N/P ratio indicates that *T. distichum* and *T. ascendens* have good adaptability to environmental changes [52]. The correlation between N and P content may come from the basic metabolic activities of land plants, such as photosynthesis and respiration [26].

### 3.3. The Relationship between Growth and C, N, and P Contents

The growth indicators of *T. distichum* and *T. ascendens* showed a negative correlation with leaf C content (Table 4 and Table 5), and flooding significantly increased the C content in their leaves (Figure 3). The reason is that C is the main product of leaf photosynthesis. C accumulation can inhibit the photosynthesis and growth of plants. Nevertheless, its defense ability against the adverse environment is enhanced [53], which is conducive to the adaptation of *T. distichum* and *T. ascendens* to water-level changes in the hydro-fluctuation zone of the TGR region. During the growing season, the N and P elements of *T. distichum* are mainly stored in the leaves [54], because the leaves of trees need more N- and P-rich substances (amino acids, transport proteins, and enzymes) to participate in metabolic activities (photosynthesis and respiration) [55]. The growth indices are positively correlated with the leaf N and P content of *T. distichum*, proving N and P’s key role in providing nutrients during plant growth. The increase in N and P content promotes the construction of plant bodies and increases plant biomass. At the same time, as with a large number of elements, N and P are very important for biochemical functions in organisms and have relatively high internal stability [27]. Therefore, in increasing biomass, N and P must be constantly replenished to maintain stability. This also leads to an increase in N and P content as the plant grows. So far, our second hypothesis has been well confirmed.

Our results showed that *T. distichum* and *T. ascendens* had higher N and P contents in photosynthetic organs (leaves) than in non-photosynthetic organs (branches and fine roots). Within an appropriate range, higher N and P content in the leaves leads to a greater net photosynthetic rate, faster growth rate, and stronger resource competition [56]. Higher N and P contents in the leaves promote the synthesis of carbohydrates needed for growth [57] and ensure the healthy growth of *T. distichum*. Therefore, N (the main element that constitutes protein) and P (the main element that constitutes nucleic acid) acquisition is essential for growth [58]. However, different results were shown in *T. ascendens*. The growth indices were negatively correlated with the leaf N and P content. The possible reason is that the concentration of N and P in *T. ascendens* leaves was diluted [59]. Since July is the vigorous period of plant growth, *T. ascendens* can carry out sufficient photosynthesis and rapid metabolism under sufficient light and rainfall conditions, promoting the plant’s vegetative organ construction. The carbohydrates produced by photosynthesis in the leaves of *T. ascendens* were not transported to other parts in time, thus diluting the N and P concentration, but *T. ascendens* still proliferated. This also confirmed part of our third hypothesis. Compared with thick roots, fine roots are more complex in structure, have higher physiological activity, and are in a state of constant renewal. The enzyme system in the cell and respiration and metabolism were active [60]. The growth of *T. distichum* and *T. ascendens* was positively correlated with the N and P content in fine roots, and more N and P elements in fine roots can promote rapid growth of fine roots, absorb more nutrients from the soil, and provide raw materials for the synthesis of proteins, nucleic acids, and other substances needed for the rapid growth of plants [61].

## 4. Materials and Methods

### 4.1. Study Site and Experimental Materials

**Figure 5:**
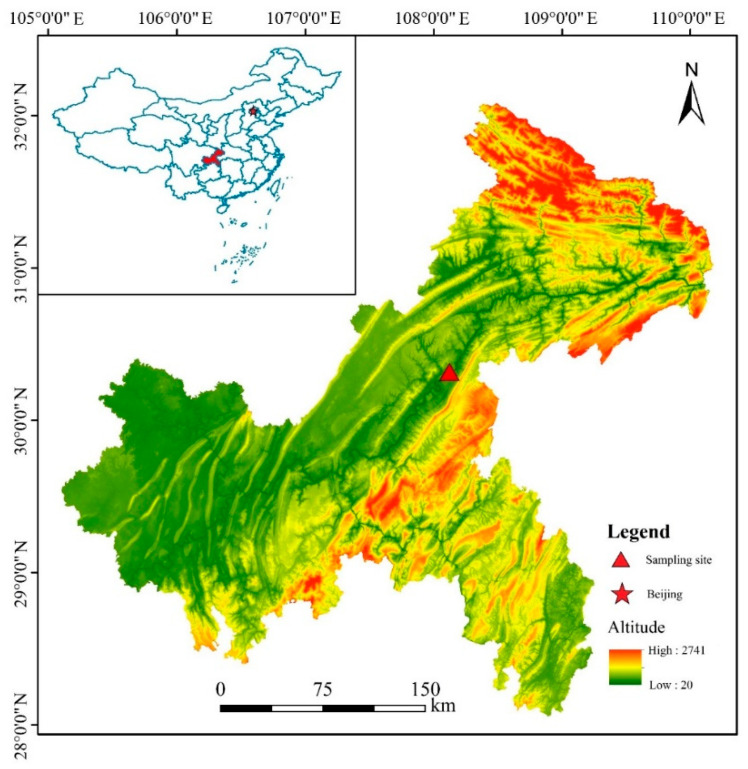
Location of sample site in the Three Gorges Dam Reservoir, China.

The study site (107°32′–108°14′ E, 30°03′–30°35′ N) was located in the Ruxi River basin in Gonghe village of Shibao Township, Zhong County, Chongqing municipality of China (Figure 5). The Ruxi River is one of the largest tributaries of the TGR. The basin is characterized by a subtropical southeast monsoonal climate [1,3,62,63,64,65]. The average annual temperature is 18.2 °C [2,4,62], and the frost-free period is 341 days. The annual sunshine hours are 1327.5, with a sunshine rate of 29% [64,65]. The total solar radiation energy is 83.7 × 4.18 kJ/cm^2^. The annual precipitation is 1200 mm, and the relative humidity is 80% [5]. The weathering of rocks is shallow, and the soil maturation degree is low. This has led to serious soil and water erosion in the less-vegetated riparian zone of the Ruxi River. To conduct in situ experimentation to rehabilitate the vegetation of the hydro-fluctuation zone of the TGR, 2 year old *T. distichum* and *T. ascendens* saplings were planted at this site at an altitude of 165–175 m in March 2012, and both species were planted in an alternating pattern with a spacing of 1 m × 1 m.

### 4.2. Sample Collection

According to the difference in winter flooding intensity, the study site was divided into three transects; 165 m (DS), 170 m (MS), and 175 m (SS) [10]. In situ sampling was carried out at the study site in July 2019. Fifty (25 each) similar and healthy *T. distichum* and *T. ascendens* were randomly selected at each elevation to sample the leaves, branches, and fine roots. We used high branch shears to collect mature leaves and branches of *T. distichum* and *T. ascendens* in four directions in the upper–middle layers of the canopy. The leaves and branches were mixed evenly and sealed in a ziplock bag. Fine roots (d ≤ 2 mm) were collected using a flat shovel, excavating the fine roots of *T. distichum* and *T. ascendens* in a radius of 0.25 m in the sample square, carefully cleaning the soil and impurities on the surface of the root system, and finally mixing the collected root system and placing the quartered part into a ziplock bag. At the same time as sampling, a height measuring rod was used to measure the plant’s height, a tape measure was used to measure the crown width of the plant, and a vernier caliper was used to measure the plant’s basal diameter and diameter at breast height (DBH).

### 4.3. Measurement of Element Content

All samples were refrigerated and transported to the laboratory, cleaned with tap water and deionized water, and placed in an oven. After 30 min of drying at 105 °C, the samples were dried to a constant weight at 65 °C. Plant samples were crushed by a Lech MM400 Ball Mill (Haan, Germany) to measure the C, N, and P contents. All samples’ C and N contents were measured using an element analyzer (Elementar Vario EL, Langenselbold, Germany). The P contents were digested by a microwave digestion apparatus (SPEEDWAVE MWS-4, Eningen, Germany) and then measured using an inductively coupled plasma emission spectrometer (ICP-OES, Thermo Fisher ICAP 6300, Northants, UK).

### 4.4. Data Analysis

All statistical analyses were performed using SPSS 25.0 and Microsoft 2019 software. One-way ANOVA followed by Tukey’s test to determine significant differences at the *p* < 0.05 level was used to analyze the effects of submergence treatment on plant growth and C, N, and P contents and stoichiometry in various parts of plants. Two-way ANOVA was used to analyze the effects of submergence treatment, different organs, and their interactions on C, N, and P contents, as well as the stoichiometry of *T. distichum* and *T. ascendens*. The independent sample *t*-test was used to analyze the differences in C, N, and P content and stoichiometry between different species under the same submergence treatment. The Pearson correlation was used to analyze the connection between the growth conditions and the C, N, and P contents of *T. distichum* and *T. ascendens*, as well as the correlation between the content of N and P in various plant organs. All images were drawn with Origin 9.0 software. Plant C, N, and P element concentrations were calculated by dry weight (g/kg), and all C/N/P ratios were calculated by mass ratio.

## 5. Conclusions

The results showed that the C, N, and P ecological stoichiometric characteristics and growth of *Taxodium distichum* and *Taxodium ascendens* differed under the three submergence treatments after years of periodic flooding. Flooding significantly affected their growth but did not endanger their health. Our findings also showed that the nutrient allocation strategies of the two species were similar. The submergence treatments, different organs, and their interactions significantly affected both species in terms of improving their stress resistance by increasing their leaves’ carbon content and coordinating the nitrogen and phosphorus contents in their organs to maintain normal functions under submergence conditions. When the submergence depth increased, the *T. ascendens* transferred more N and P elements from the fine roots to the leaves for adaptation to the periodic flooding. However, the N/P ratio in all organs of *T. distichum* and *T. ascendens* was lower than the critical ratio of 14 in all treatments, indicating that the N element may restrict the growth of the two species. In contrast, the N and P content in all organs of the two species were higher than the national average level, making up for the limitation of the N element. In summary, *Taxodium distichum* and *Taxodium ascendens* can respond positively to submergence and coordinate the allocation of nutrient elements among various organs to better adapt to the complex hydrological conditions in the hydro-fluctuation zone of the Three Gorges Reservoir.

## Figures and Tables

**Table 1:** Growth parameters of *Taxodium distichum* and *Taxodium ascendens* under different submergence treatments.

Growth Parameters	*Taxodium distichum*			*Taxodium ascendens*		
	SS	MS	DS	SS	MS	DS
Height (m)	6.87 ± 0.07 a	5.59 ± 0.01 b	5.41 ± 0.01 c	6.27 ± 0.04 a	5.73 ± 0.03 b	5.46 ± 0.04 c
Crown area (m^2^)	8.01 ± 0.17 a	6.45 ± 0.11 b	4.79 ± 0.28 c	7.23 ± 0.20 a	5.81 ± 0.21 b	5.03 ± 0.08 c
Basal diameter (mm)	104.95 ± 0.99 a	94.58 ± 1.91 b	86.51 ± 1.71 c	100.16 ± 3.22 a	94.35 ± 2.20 ab	86.98 ± 2.34 b
DBH (mm)	73.12 ± 1.99 a	65.00 ± 0.56 b	56.85 ± 0.21 c	79.37 ± 2.30 a	71.58 ± 2.78 ab	66.35 ± 2.74 b

Note: Values are means ± SE. The values with different lowercase letters are significantly different at *p* < 0.05 according to Tukey’s test. DBH—diameter at breast height. DS—deep submergence; MS—moderate submergence; SS—shallow submergence.

**Table 2:** Effects of submergence treatment and different organs on carbon, nitrogen, and phosphorus stoichiometry of *Taxodium distichum*.

Indexes	Submergence		Organ		Submergence × Organ	
	*F*	*p*	*F*	*p*	*F*	*p*
C content	2.539	0.093	18.826	0.000 ***	5.955	0.001 ***
N content	12.563	0.000 ***	1493.660	0.000 ***	5.331	0.002 **
P content	44.706	0.000 ***	1545.718	0.000 ***	60.428	0.000 ***
C/N ratio	46.386	0.000 ***	1563.755	0.000 ***	37.627	0.000 ***
C/P ratio	37.580	0.000 ***	2585.621	0.000 ***	186.738	0.000 ***
N/P ratio	7.390	0.002 **	177.072	0.000 ***	24.142	0.000 ***

Note: ***, ** indicate significance at the levels of 0.001, 0.01, and 0.05 among different submergence treatments, different organs, and their interaction.

**Table 3:** Effects of submergence treatment and different organs on carbon, nitrogen, and phosphorus stoichiometry of *Taxodium ascendens*.

Indexes	Submergence		Organ		Submergence × Organ	
	*F*	*p*	*F*	*p*	*F*	*p*
C content	6.005	0.006 **	10.217	0.000 ***	1.134	0.356
N content	32.213	0.000 ***	1417.414	0.000 ***	14.366	0.000 ***
P content	1.748	0.189	361.708	0.000 ***	49.614	0.000 ***
C/N ratio	29.012	0.000 ***	1607.768	0.000 ***	12.423	0.000 ***
C/P ratio	22.472	0.000 ***	1996.119	0.000 ***	49.289	0.000 ***
N/P ratio	31.091	0.000 ***	190.933	0.000 ***	38.294	0.000 ***

Note: ***, ** indicate significance at the levels of 0.001, 0.01, and 0.05 among different submergence treatments, different organs, and their interaction.

**Table 4:** Correlation analysis of plant height, crown area, basal diameter, and DBH with the C, N, and P content in various organs of *Taxodium distichum*.

Growth Parameters	C in Fine Root	N in Fine Root	P in Fine Root	C in Leaf	N in Leaf	P in Leaf	C in Branch	N in Branch	P in Branch
Height	0.373	0.588 *	0.904 **	−0.785 **	0.712 **	0.499	−0.077	0.203	−0.315
Crown area	0.435	0.516 *	0.642 **	−0.678 **	0.685 **	0.163	−0.221	0.505	0.069
Basal diameter	0.465	0.685 **	0.649 **	−0.578 *	0.553 *	0.289	−0.139	0.480	0.024
DBH	0.537 *	0.524 *	0.627 *	−0.598 *	0.669 **	0.217	−0.087	0.533 *	0.082

Note: ** and * indicate significance at the levels of 0.01 and 0.05, respectively.

**Table 5:** Correlation analysis of plant height, crown area, basal diameter, and DBH with the C, N, and P content in various organs of *Taxodium ascendens*.

Growth Parameters	C in Fine Root	N in Fine Root	P in Fine Root	C in Leaf	N in Leaf	P in Leaf	C in Branch	N in Branch	P in Branch
Height	0.053	0.785 **	0.767 **	−0.534 *	−0.080	−0.662 **	−0.248	−0.115	−0.134
Crown area	0.045	0.697 **	0.746 **	−0.629 *	−0.087	−0.687 **	−0.262	−0.185	−0.066
Basal diameter	−0.201	0.469	0.627 *	−0.594 *	−0.314	−0.699 **	−0.051	−0.196	0.188
DBH	−0.080	0.457	0.549 *	−0.427	−0.188	−0.620 *	−0.198	−0.094	0.032

Note: ** and * indicate significance at the levels of 0.01 and 0.05, respectively.

## Data Availability

The data presented in this study are available in the figures and tables.
